# The role of intestinal microbial metabolites in polycystic ovary syndrome: mechanistic insights and intervention prospects

**DOI:** 10.3389/fendo.2026.1886749

**Published:** 2026-09-10

**Authors:** Ye Gao, Linlin Fan, Rui Ma, Linling Wu

**Affiliations:** 1 First Teaching Hospital of Tianjin University of Traditional Chinese Medicine, Tianjin, China; 2 National Clinical Research Center for Chinese Medicine, Tianjin, China

**Keywords:** gut microbial metabolites, gut-ovary axis, hyperandrogenism, insulin resistance, polycystic ovary syndrome

## Abstract

**Background:**

Polycystic ovary syndrome (PCOS) is a prevalent endocrine-metabolic disorder among women of reproductive age, defined by the core features of hyperandrogenism, ovulatory dysfunction, and polycystic ovarian morphology. PCOS is frequently complicated by insulin resistance, obesity, dyslipidemia, and chronic low-grade inflammation, which substantially impair patients’ reproductive health, metabolic homeostasis, and quality of life. Although the etiology of PCOS remains incompletely elucidated, accumulating evidence indicates that gut microbiota and their metabolites participate in the pathogenesis and progression of PCOS via metabolic, immune, and neuroendocrine pathways. Focusing on the gut-ovary axis, this review systematically summarizes the molecular mechanisms by which gut microbial metabolites mediate PCOS-related pathological processes, as well as potential intervention strategies.

**Methods:**

This article is a narrative review. To ensure comprehensive literature coverage, we searched the PubMed, Embase, Web of Science, and Cochrane Library databases from their inception to June 30, 2026. Search terms included polycystic ovary syndrome, PCOS, gut microbiota, gastrointestinal microbiota, microbial metabolites, short-chain fatty acids, bile acids, lipopolysaccharide, branched-chain amino acids, tryptophan metabolism, gut-ovary axis, probiotics, prebiotics, postbiotics, and fecal microbiota transplantation. Original basic research, clinical observational studies, randomized controlled trials, systematic reviews, meta-analyses, and clinical guidelines related to PCOS and gut microbiota were prioritized for inclusion. Content lacking clear support from primary literature, with evidence limited to other disease models, or involving excessive mechanistic extrapolation was either excluded or interpreted conservatively.

## 1 Introduction

Polycystic ovary syndrome (PCOS) is one of the most common endocrine and metabolic disorders in women of reproductive age, with a global prevalence as high as 6%–13% (1), and it is most prevalent among women aged 15–49 years. Its core clinical features include hyperandrogenism, ovulatory dysfunction, and polycystic ovarian changes, often accompanied by insulin resistance, obesity, lipid metabolism disorders, and mood disorders, which seriously impair patients’ reproductive health and long-term quality of life (2). At present, the pathophysiological mechanism of PCOS has not been fully elucidated. Traditionally, PCOS has been regarded as a gynecological endocrine or metabolic disorder in isolation. Therefore, although current clinical intervention strategies (e.g., hormonal modulation and insulin sensitization) can partially relieve symptoms, it is difficult to achieve etiological treatment and long-term disease management. Accordingly, exploring the systemic and root pathogenesis of PCOS has become an urgent scientific demand in this field.

Qi et al. (3) showed that transplantation of gut microbiota from PCOS patients to germ-free mice successfully induced PCOS-like phenotypes (including ovulatory dysfunction, hormonal disturbances, and polycystic ovarian morphology), indicating that gut microbial dysbiosis contributes causally to PCOS pathogenesis rather than acting as a mere concomitant epiphenomenon. However, this causal finding is restricted to preclinical germ-free mouse models, with no conclusive causal data yet available from large-scale human studies. Further mechanistic studies in animal models identified a microbiota–bile acid–IL-22 axis and reported that IL-22 or secondary bile acid supplementation was associated with improved ovarian function in these models. In addition to the above findings, Feng et al. (4) reported that in a PCOS rat model, sodium butyrate (NaBu) treatment restored estrous cycles and ovulation, reduced serum testosterone and LH levels, and increased estradiol and progesterone levels. Mechanistic studies suggested that NaBu may exert its regulatory effects through the gut–brain–ovary axis, modulating ovarian steroidogenic factor expression and improving granulosa cell function.

Together, these studies have shown that dysregulated gut microbiota can act as key biological messengers across anatomical distances through its abundant metabolites, such as short-chain fatty acids (SCFAs), branched-chain amino acids (BCAAs), bile acids (BAs), lipopolysaccharide (LPS), and tryptophan metabolites. Through blood circulation, immune-inflammatory and neuroendocrine networks, the ovarian microenvironment, steroidogenesis, follicle development, and ovulation are regulated remotely and precisely. This communication network, mediated by gut microbiota and its metabolites, systematically regulates ovarian function, which can be summarized as the gut-ovary axis (5).

## 2 Microbial composition

Gut microbiota is a complex microecosystem composed of bacteria, fungi, and viruses (bacteriophages). Bacteria are the core functional groups, while fungi and viruses are important regulatory components, which jointly participate in the regulation of host metabolism, immunity, and endocrine homeostasis (6). The gut microbiota plays a role in regulating sex hormone homeostasis, energy metabolism balance, and maintenance of intestinal mucosal barrier integrity (6). When this microecological balance is disrupted, the enrichment of pathogenic bacteria and abnormal metabolites can induce systemic inflammation, thereby disrupting the function of the gut-ovarian axis and aggravating the core pathological features of PCOS, such as hyperandrogenism and insulin resistance (6, 7).

### 2.1 Bacteria

The composition and diversity of gut microbiota are closely linked to the pathogenesis of PCOS, and current evidence supports a bidirectional regulatory relationship between the two (7). Firmicutes and Bacteroidetes are the dominant bacterial phyla in the human gut, collectively accounting for approximately 90% of fecal microbiota in healthy individuals, and serve as the primary producers of intestinal short-chain fatty acids (SCFAs) (7).

Early studies confirmed that PCOS-associated intestinal dysbiosis is primarily characterized by an imbalance between beneficial and pathogenic bacteria, as well as reduced overall microbial diversity (7). With technological advances, contemporary microbiome research on PCOS has gradually shifted from descriptive analyses of global microbial community structure to functional annotation of specific bacterial species and strains. Meanwhile, integrated multi-omics approaches including metagenomics, metabolomics, and transcriptomics enable more precise delineation of the functional roles of gut microbiota in PCOS onset (8).

Large-scale clinical cohort studies and systematic reviews consistently demonstrate significant structural abnormalities in the gut microbiota of PCOS patients, manifested as increased harmful bacteria and decreased beneficial bacteria (7). In treatment-naïve PCOS patients, the abundances of Desulfovibrio and Bacteroides are elevated; by contrast, functional commensals that maintain intestinal barrier integrity and regulate systemic metabolic homeostasis are markedly reduced, including core SCFA-producing taxa such as *Roseburia*, *Faecalibacterium*, and Lachnospiraceae, as well as *Akkermansia muciniphila*, which exerts intestinal barrier-protective effects (9).

Among these functionally beneficial bacteria, Roseburia abundance correlates with clinical phenotypes of PCOS and is negatively associated with insulin resistance and hyperandrogenism (8). Further shotgun metagenomic sequencing studies verify that compared with healthy individuals, both obese and lean PCOS patients exhibit significantly reduced intestinal abundance of *Faecalibacterium prausnitzii*, whereas certain Bacteroides species show increased abundance (8).

*Faecalibacterium prausnitzii* is a core intestinal short-chain fatty acid (SCFA)-producing bacterium and a primary butyrate-synthesizing microbe in the human gut. A growing body of evidence from animal models and human cross-sectional studies indicates that the abundance of this bacterium is significantly lower in the gut of patients with polycystic ovary syndrome (PCOS) compared with healthy controls, and this reduction is closely associated with chronic low-grade inflammation and abnormal metabolic parameters in these patients (8). As key metabolites of this bacterium, SCFAs have been demonstrated in both *in vivo* and *in vitro* experiments to effectively maintain the structural integrity of the intestinal barrier (10, 11). Therefore, it is hypothesized that the depletion of *F. prausnitzii* may exacerbate intestinal barrier impairment in patients with PCOS, although large-scale interventional clinical evidence in humans remains lacking. Furthermore, human observational studies have established an association between gut dysbiosis and hyperandrogenic phenotypes in PCOS, but the causal relationship between the two has not yet been fully elucidated.

Clinical studies further indicate that PCOS patients with lower *F. prausnitzii* abundance present with more severe disease and higher risk of metabolic complications (8). During the progression of PCOS-associated dysbiosis, SCFA-producing beneficial bacteria (e.g., Roseburia and *Faecalibacterium*) decline significantly, while potentially pathogenic *Escherichia*/Shigella strains increase markedly. These bidirectional structural alterations may collectively induce intestinal barrier dysfunction, trigger persistent chronic low-grade inflammation, and ultimately contribute to the development and progression of PCOS (7).

Among the abnormally enriched taxa, Bacteroides and Desulfovibrio have received particular attention in PCOS pathophysiology research. Animal experiments show that their abundances may change with PCOS progression, suggesting involvement in core pathological processes. Mechanistic studies indicate that β-glucuronidase secreted by intestinal Bacteroides may modulate the enterohepatic circulation of sex steroids, but its direct correlation with androgen levels remains controversial (12). Furthermore, mechanistic evidence demonstrates that hydrogen sulfide produced by Desulfovibrio disrupts the intestinal epithelial barrier and induces systemic chronic low-grade inflammation. Notably, inflammatory cytokines have been confirmed to upregulate the expression and activity of cytochrome P450 17A1 (CYP17A1), a key enzyme in ovarian androgen synthesis (13). Based on these findings, we propose that Desulfovibrio may indirectly regulate ovarian androgen synthesis by eliciting inflammatory responses.

Moreover, hyperandrogenism—the core clinical feature of PCOS—is not caused by unidirectional microbial abnormalities; instead, it forms a bidirectional regulatory loop with gut dysbiosis. On the one hand, gut dysbiosis exacerbates PCOS pathology via metabolic disturbance, chronic inflammation, and impaired androgen clearance. On the other hand, hyperandrogenism remodels gut microbial structure and correlates with reduced microbial diversity. Animal experiments demonstrate that prenatal and early postnatal hyperandrogen exposure exerts long-term effects on gut microbial structure and metabolic function, thereby increasing PCOS susceptibility (14).

The gut microbiota of PCOS patients undergoes characteristic dynamic shifts with disease progression and clinical interventions. Metformin, a first-line insulin sensitizer, significantly modulates gut microbial composition and diversity and ameliorates dysbiosis in PCOS (15). However, clinical evidence has not reached a consensus on the effect of 3–6 months of continuous combined oral contraceptive use on gut microbiota in PCOS patients (16). The differential modulatory effects of therapeutic agents on gut microecology represent a major source of heterogeneity in current clinical research on PCOS gut microbiota. Meanwhile, the strong interindividual variability of gut microbiota further complicates investigations into the microbial mechanisms of PCOS (7).

### 2.2 Fungi and viruses

Gut microbial dysbiosis is a multidimensional ecosystem disturbance that involves not only bacteria but also alterations in fungal microbiota (mycobiota) and viral components (mainly bacteriophages) in PCOS, which together constitute a complex gut-ovary regulatory network (6).

Studies have shown that the composition of intestinal fungal communities is significantly altered in PCOS patients. For example, Chen et al. found that PCOS patients exhibited significantly increased abundance of Ascomycota and yeast-associated fungal families and genera, alongside reduced abundance of Basidiomycota, *Trichophyton*, *Aspergillus*, and other genera, indicating structural disruption of the gut fungal community (17). More importantly, a study by Wu et al. provided functional evidence supporting a pathogenic role of gut fungi in PCOS using rodent models. This study found that the abundance of *Aspergillus tubingensis* is significantly increased in the gut of PCOS patients. The secondary metabolite AT-C1 secreted by this fungal strain acts as an endogenous aryl hydrocarbon receptor (AhR) antagonist by competitive inhibition of AhR signaling, which in turn blocks the secretion of interleukin-22 (IL-22) by type 3 innate lymphoid cells (ILC3). In animal models, this pathway directly induces the core phenotypes of PCOS, such as hyperandrogenism and ovulatory dysfunction. This study, for the first time, identified that specific gut fungi and their active metabolites could play a key role in the pathogenesis of PCOS by directly regulating host immunity, providing a new mechanism beyond bacteria for the gut-ovary axis (18).

Viruses (mainly bacteriophages) also undergo characteristic changes in PCOS. The enteric virome is characterized by reduced diversity and disrupted composition. For example, the abundance of phage families such as Siphoviridae was significantly reduced, while families such as Quimbyviridae were relatively enriched. Phage dysbiosis may alter the abundance and function of their bacterial hosts (e.g., *Bacteroides*), thereby indirectly contributing to the onset and progression of core metabolic and immune disturbances in PCOS, including insulin resistance, hyperandrogenism, and chronic low-grade inflammation (19).

## 3 Core molecular mechanisms of the gut-ovary axis

### 3.1 Intestinal barrier damage, LPS translocation, and the TLR4/NF-κB inflammatory pathway

Intestinal barrier dysfunction serves as a critical link between gut dysbiosis and chronic low-grade inflammation in PCOS. Gut microbiota imbalance downregulates tight junction protein expression, disrupts mucus layer homeostasis, and increases intestinal permeability (20). Following barrier impairment, pathogen-associated molecular patterns such as lipopolysaccharide (LPS) translocate into the systemic circulation, causing metabolic endotoxemia. Upon binding to lipopolysaccharide-binding protein, LPS activates Toll-like receptor 4 (TLR4) with the assistance of CD14 and MD-2, which in turn triggers inflammatory signaling cascades including nuclear factor-κB (NF-κB) and mitogen-activated protein kinase (MAPK), and promotes the release of proinflammatory cytokines such as tumor necrosis factor-α (TNF-α), interleukin-6 (IL-6), and interleukin-1β (IL-1β) (20, 21).

Chronic low-grade inflammation aggravates PCOS pathology through multiple mechanisms. First, inflammatory cytokines interfere with physiological phosphorylation of insulin receptor substrates, inhibit phosphatidylinositol 3-kinase/protein kinase B (PI3K/Akt) signaling, and promote insulin resistance. Second, the inflammatory microenvironment impairs the function of ovarian granulosa cells and theca cells, disrupting folliculogenesis and steroidogenesis. Some animal and *in vitro* studies suggest that LPS-TLR4/NF-κB signaling may alter the expression of steroidogenic regulators such as steroidogenic acute regulatory protein (StAR) and CYP17A1, but direct evidence in human PCOS ovarian tissue requires further validation (21, 22).

### 3.2 Short-chain fatty acids, G protein-coupled receptors, and epigenetic regulation

Short-chain fatty acids (SCFAs)—mainly acetate, propionate, and butyrate—are key metabolites generated by gut microbial fermentation of dietary fiber. Reduced abundance of SCFA-producing bacteria (e.g., *Roseburia*, *Faecalibacterium*, and Lachnospiraceae) is frequently reported in PCOS, although fecal and circulating SCFA concentrations are inconsistent across studies (4, 23).

SCFAs regulate PCOS-related metabolism and inflammation via multiple pathways. First, butyrate is a primary energy substrate for colonic epithelial cells and supports tight junction protein expression and intestinal barrier integrity. Second, SCFAs activate receptors including free fatty acid receptor 2/G protein-coupled receptor 43 (FFAR2/GPR43), FFAR3/GPR41, and hydroxycarboxylic acid receptor 2 (HCAR2), stimulating secretion of enteroendocrine hormones such as glucagon-like peptide-1 (GLP-1) and peptide YY (PYY), thereby modulating appetite, glucose metabolism, and insulin sensitivity (4, 23). Third, SCFAs (notably butyrate) exert histone deacetylase (HDAC) inhibitory activity and regulate inflammation- and metabolism-related gene expression via epigenetic mechanisms (4).

Accumulating studies in polycystic ovary syndrome (PCOS) models have demonstrated that butyrate supplementation or enhancement of endogenous short-chain fatty acid (SCFA) production can ameliorate ovulatory dysfunction and hyperandrogenic phenotypes (4, 24). However, whether these beneficial effects stem from the direct regulatory actions of SCFAs on ovarian tissue remains a critical evidence gap in this field.

At the mechanistic level, millimolar concentrations of butyrate modulate the expression of key steroidogenic enzymes, including steroidogenic acute regulatory protein (StAR), CYP19A1, and CYP17A1, via histone deacetylase (HDAC) inhibition, but such concentrations greatly exceed physiological levels (4, 25). Under physiological conditions, SCFAs exert biological effects mainly through G protein-coupled receptors 41/43 (GPR41/GPR43), with a half-maximal effective concentration (EC_50_) in the micromolar range. However, the expression levels and functional activity of these receptors in ovarian tissue have not been fully elucidated (26, 27). Notably, the improved ovarian function observed in animal models is frequently accompanied by simultaneous alleviation of insulin resistance and chronic low-grade inflammation, making it impossible to rule out secondary effects mediated by systemic metabolic improvements (28, 29).

Taken together, the ameliorative effects of endogenous SCFAs on reproductive phenotypes in PCOS are more likely dominated by indirect regulatory pathways: SCFAs promote glucagon-like peptide-1 (GLP-1) secretion by activating intestinal GPR43 to improve insulin resistance, and suppress NF-κB signaling to attenuate chronic inflammation, thereby indirectly reducing ovarian androgen synthesis. In contrast, direct regulation of ovarian steroidogenesis may only occur under high-concentration pharmacological exposure (28). The actual *in vivo* contribution of endogenous SCFAs to ovarian function remains to be further verified by detection of local ovarian SCFA concentrations and targeted delivery studies.

### 3.3 Bile acids, FXR/TGR5 signaling, and IL-22-mediated immunomodulation

Bile acids participate not only in lipid digestion and absorption but also act as pivotal metabolic and immune signaling molecules. Primary bile acids synthesized in the liver enter the intestine and undergo deconjugation, oxidation, epimerization, and 7α-dehydroxylation mediated by gut microbiota, generating diverse secondary bile acids. Altered gut microbial composition reshapes the bile acid profile, thereby modulating signaling through bile acid receptors such as farnesoid X receptor (FXR) and Takeda G protein-coupled receptor 5 (TGR5) (30).

FXR and TGR5 are broadly involved in glucose and lipid metabolism, energy homeostasis, inflammation regulation, and intestinal barrier function. PCOS-related studies suggest that aberrant bile acid metabolism is associated with insulin resistance, hyperandrogenism, and chronic inflammation (30). Some animal studies show that modulating bile acid composition ameliorates PCOS-like phenotypes via IL-22-dependent pathways. Interleukin-22 (IL-22) preserves intestinal barrier function and regulates mucosal immunity; its deficiency may exacerbate inflammation and metabolic dysfunction (3).

Notably, individual bile acid species exert distinct, sometimes opposing, receptor activities and tissue effects. Therefore, “elevated primary bile acids” or “reduced secondary bile acids” cannot be generalized as a uniform pathogenic pattern. Future studies should integrate targeted bile acid metabolomics, functional gut microbiota profiling, and host receptor signaling analysis to clarify the roles of specific bile acid molecules in PCOS (30).

### 3.4 Branched-chain amino acids, mTORC1 signaling, and insulin resistance

Branched-chain amino acids (BCAAs)—leucine, isoleucine, and valine—are metabolites closely linked to insulin resistance. Metabolomic studies reveal elevated circulating BCAA levels in PCOS patients, which correlate with insulin resistance, obesity, and metabolic abnormalities. BCAAs originate from dietary intake and gut microbial metabolism, and their homeostasis is also governed by the catabolic capacity of the liver, skeletal muscle, and adipose tissue (31).

Mechanistically, excess BCAAs activate the mammalian target of rapamycin complex 1/ribosomal S6 kinase 1 (mTORC1/S6K1) pathway, promoting aberrant serine phosphorylation of insulin receptor substrates and attenuating insulin signal transduction. Insulin resistance and secondary hyperinsulinemia further stimulate ovarian androgen synthesis and suppress hepatic sex hormone-binding globulin (SHBG) production, worsening hyperandrogenism (31, 32).

Current evidence linking BCAA dysregulation to PCOS is largely correlational and extrapolated from metabolic disease mechanisms. Sufficient evidence is lacking regarding whether BCAAs directly act on human ovarian theca cells to promote androgen synthesis. A more conservative interpretation is that abnormal BCAA metabolism indirectly impairs ovarian endocrine function by exacerbating insulin resistance (31).

### 3.5 Tryptophan metabolism, AhR signaling, and neuroimmunomodulation

Tryptophan, an essential amino acid, is metabolized by the host and gut microbiota into a spectrum of bioactive products, including indole derivatives, kynurenine pathway metabolites, and serotonin-related molecules. Gut microbiota convert tryptophan into multiple aryl hydrocarbon receptor (AhR) ligands, such as indole-3-propionic acid, indole-3-acetic acid, and indole-3-carboxaldehyde. These metabolites maintain intestinal barrier integrity, regulate mucosal immunity, and constrain inflammation (33).

AhR signaling modulates IL-22 production, Th17/Treg balance, and intestinal epithelial barrier homeostasis. PCOS-associated dysbiosis may reduce beneficial indole metabolites, weakening AhR-mediated immune protection and promoting chronic low-grade inflammation (33, 34). Meanwhile, shunting of tryptophan metabolism toward the kynurenine pathway may contribute to inflammation, oxidative stress, and neuropsychiatric symptoms. The high prevalence of anxiety and depression in PCOS may be partially mediated by interactions among gut microbiota, tryptophan metabolism, and the central nervous system (34).

Research on tryptophan metabolism dysregulation in PCOS remains in its early stages. The therapeutic potential of AhR ligand supplementation, kynurenine pathway modulation, and NAD^+^ metabolism intervention requires further validation in animal models and human studies (33).

## 4 Systemic inflammation

Gut microbial dysbiosis underpins the persistent state of chronic low-grade inflammation observed in patients with PCOS. This inflammation serves as the key pathological bridge in the gut-ovary axis, linking distal gut disturbances to local ovarian dysfunction. Its core pathological effects are centered on two key targets: the ovary and adipose tissue (35).

### 4.1 Local immune microenvironment of the ovary

Ovarian inflammation is prevalent in PCOS patients. The initiation of this local inflammation is partly attributed to increased translocation of gut-derived pathogen-associated molecular patterns (PAMPs, e.g., lipopolysaccharide [LPS]). These molecules upregulate the local ovarian expression of Toll-like receptor 4 (TLR4) and activate downstream NF-κB signaling pathways, which in turn drives the transcription of proinflammatory cytokines including IL-1β and TNF-α and initiates a cascade of inflammatory signaling (29). Besides soluble PAMPs, extracellular vesicles (EVs) derived from both the gut microbiota and adipose tissue act as particulate mediators that play a key role in inflammatory signal transmission. These EVs carry specific miRNAs, proteins, and lipid components, and can remotely target the ovary via the circulation to directly regulate signaling networks in recipient cells, constituting another early inflammatory initiation pathway independent of the classical LPS-TLR4 pathway (8, 36).

Ovarian secretion of C-C motif chemokine ligand 2 (CCL2/MCP-1) is significantly increased under inflammatory stimulation. The upregulation of MCP-1 is regulated by multiple mechanisms. Gut microbial dysbiosis not only triggers inflammation via LPS translocation, but also reduces the production of beneficial metabolites such as short-chain fatty acids (SCFAs, e.g., butyrate), weakens their anti-inflammatory and barrier-protective effects, and alters bile acid metabolism, disrupting the FXR/TGR5 receptor-mediated balance between metabolism and inflammation. These alterations collectively drive chronic low-grade inflammation, insulin resistance (IR), and hyperandrogenism, creating a microenvironment that sustains MCP-1 expression (35, 37). Elevated MCP-1 concentrations trigger monocyte migration and activation, which in turn increases inflammatory factor production, and the inflammatory response further damages ovarian tissue. In addition, ovarian MCP-1 promotes macrophage infiltration into periovarian adipose tissue, linking ovarian inflammation to abdominal obesity and metabolic disorders. Collectively, MCP-1 plays a pivotal role in chronic low-grade inflammation (37, 38).

Local proinflammatory cytokines are key pathological mediators driving disruption of the intraovarian immune-endocrine microenvironment in women with polycystic ovary syndrome (PCOS). Women with PCOS consistently exhibit chronic low-grade inflammation, yet circulating and intraovarian cytokine concentrations vary across individuals, modulated by factors including obesity status, severity of insulin resistance, phenotypic subtypes, and study population characteristics (13). IL-12, a critical upstream regulator of type 1 immune responses, drives T helper type 1 (Th1) polarization and interferon-γ production, thereby amplifying cell-mediated inflammatory cascades (39, 40). By potentiating Th1-skewed immune deviation, IL-12 contributes to the establishment of a proinflammatory ovarian microenvironment and promotes the secretion of downstream mediators, including tumor necrosis factor-α (TNF-α) and interleukin-6 (IL-6) (13, 41).

TNF-α and IL-6 are well-recognized inflammatory mediators that link systemic metabolic dysfunction to ovarian impairment in PCOS. TNF-α impairs insulin signaling primarily by inducing serine phosphorylation of insulin receptor substrate-1 (IRS-1), which inhibits its tyrosine phosphorylation and subsequent downstream signal transduction, thereby contributing to the development and progression of both peripheral and local ovarian insulin resistance (29). Within the ovary, proinflammatory cytokines exert detrimental effects on granulosa cell function, follicular maturation, and local responsiveness to insulin and gonadotropins (13). Beyond granulosa cell dysfunction, dysregulated steroidogenesis in ovarian theca-interstitial cells represents a core driver of hyperandrogenism in PCOS. Notably, primary theca cells isolated from human polycystic ovaries retain a stable, intrinsic phenotype of excessive androgen biosynthesis even after long-term serial propagation, as demonstrated by classical *in vitro* studies (42).

Accumulating *in vitro* and *in vivo* evidence, primarily from rat theca-interstitial cell models and other steroidogenic cell types, indicates that TNF-α initiates intracellular signaling cascades mainly via the TNFR1 receptor on theca-interstitial cells, with the NF-κB and MAPK pathways serving as the core downstream axes regulating steroidogenic enzyme expression and androgen biosynthesis (42). In human ovarian theca cells, however, direct evidence for this complete signaling axis remains limited, with supporting data mostly derived from functional inhibition studies (13). These two pathways modulate CYP17A1 at both the transcriptional and post-translational levels, collectively driving excessive androgen biosynthesis. At the transcriptional level, activated ERK1/2 and p38 MAPK phosphorylate steroidogenic factor-1 (SF-1, encoded by NR5A1) and markedly enhance its transactivation activity. As the master transcription factor governing steroidogenesis, SF-1 directly binds to conserved response elements in the CYP17A1 promoter to upregulate total gene expression, thereby concomitantly increasing the basal activities of both 17α-hydroxylase and 17,20-lyase (13). Proinflammatory TNF-α is capable of triggering multiple intracellular cascades in ovarian theca-interstitial cells and modulates steroidogenic output, as documented in rodent *in vitro* models (43). By analogy to related steroid-producing cell types such as Leydig cells, TNF-α is presumed to signal through TNFR1, with downstream engagement of MAPK and NF-κB pathways (2). Nevertheless, direct experimental evidence for this complete signaling cascade within ovarian theca-interstitial cells remains sparse, and validation in human primary cells is lacking (13, 44). At the post-translational level, TNF-α-activated p38α MAPK directly phosphorylates serine/threonine residues of the CYP17A1 protein. This modification selectively and markedly enhances its 17,20-lyase activity—the rate-limiting step of ovarian androgen biosynthesis—by facilitating the interaction of the enzyme with its electron donor cytochrome P450 oxidoreductase and allosteric cofactor cytochrome b5, while exerting minimal effects on 17α-hydroxylase activity (28). This selective regulation shifts the steroidogenic pathway toward androgen biosynthesis, representing a key specific mechanism by which chronic low-grade inflammation induces hyperandrogenemia (45, 46). Nevertheless, the aforementioned complete molecular regulatory axis has mostly been demonstrated in primary rat theca cells or other steroidogenic cell types (41, 46). Direct causal evidence for TNF-α regulation of CYP17A1 transcription and enzymatic activity via the full TNFR1–NF-κB/MAPK–SF-1 pathway in human ovarian theca cells remains lacking (45, 47).

IL-6 further exacerbates insulin resistance by activating the JAK/STAT signaling pathway and subsequently inducing the expression of suppressor of cytokine signaling 3 (SOCS3) (48). Collectively, elevated proinflammatory cytokine activity contributes to granulosa cell dysfunction, aberrant folliculogenesis, local and systemic insulin resistance, and ultimately reproductive impairment in women with PCOS (47, 49). However, the causal relationships and precise molecular mechanisms linking IL-12, TNF-α, and IL-6 to dysregulated ovarian steroidogenesis remain to be further validated by targeted *in vitro* and *in vivo* functional studies (41, 47).

### 4.2 Adipose tissue inflammation

In PCOS patients with an obese phenotype, adipose tissue, especially visceral fat, acts as a key source and amplifier of systemic inflammation. Macrophage polarization is imbalanced: proinflammatory M1 macrophages increase and anti-inflammatory M2 macrophages decrease, resulting in an overall shift of the adipokine secretion profile toward a proinflammatory state (e.g., increased TNF-α and IL-6, decreased adiponectin and IL-10) (48). The influx of adipose-derived inflammatory cytokines into the circulation has multiple systemic effects. First, they act remotely on the ovary to exacerbate local inflammation and stimulate androgen synthesis in theca cells; second, by activating kinases including JNK and IKKβ, they interfere with insulin receptor signaling and directly exacerbate insulin resistance in peripheral tissues such as the liver and skeletal muscle. Third, they also promote hepatic gluconeogenesis and disrupt blood glucose homeostasis via the JAK–STAT3 pathway and other signaling cascades. Therefore, adipose tissue is not only an energy storage organ, but also an inflammatory hub linking metabolic disorders and reproductive endocrine dysfunction (50, 51).

## 5 Neuroendocrine regulation

The pathogenesis of PCOS also involves abnormal neuroendocrine regulation. Gut microbiota and their metabolites (e.g., SCFAs and tryptophan metabolites) affect central nervous system function via the gut–brain axis, and in turn regulate the hypothalamic–pituitary–ovarian (HPO) axis, forming the gut–brain–ovarian axis. This provides an integrated perspective for explaining reproductive dysfunction, emotional abnormalities, and stress responses in PCOS patients (6).

### 5.1 Direct and indirect regulation of gut metabolites on the HPO axis

Intestinal microbial metabolites such as short-chain fatty acids (SCFAs) can regulate HPO axis function via multiple mechanisms. SCFAs (e.g., butyrate) activate G protein-coupled receptors (GPR41/43) on intestinal epithelial cells, stimulating enteroendocrine cells to release gut hormones such as glucagon-like peptide-1 and peptide YY (52). In addition to regulating glucose metabolism and appetite, these hormones can also act on the hypothalamus through vagus nerve afferent signals or blood circulation. Moreover, these gut hormones affect the pulsatile secretion pattern of gonadotropin-releasing hormone (GnRH) neurons. Studies have shown that SCFA deficiency can lead to abnormal GnRH release rhythms, which in turn increases the LH/FSH ratio and disrupts follicular development and ovulation. In addition, tryptophan metabolites such as indoles can indirectly participate in GnRH release and mood regulation by modulating serotonin synthesis and metabolism (6, 33).

### 5.2 Chronic stress and HPA axis dysfunction

Chronic psychological or physiological stress can activate the hypothalamic–pituitary–adrenal (HPA) axis, elevate cortisol levels, and in turn alter gut microbiota composition and increase intestinal permeability, forming a vicious cycle of “stress–intestinal disturbance” (33, 34). Stress-induced microbiota alterations further affect tryptophan metabolism, shunting it toward the kynurenine pathway and reducing precursors for 5-hydroxytryptamine (5-HT) synthesis (28, 33). Meanwhile, microbiota-derived metabolites can also cross the blood–brain barrier to affect the central 5-HT system and HPA axis feedback regulation, which exacerbates GnRH pulse generator dysfunction and contributes to ovulatory dysfunction and mood symptoms in PCOS patients (52).

### 5.3 5-Hydroxytryptamine system

**Figure 1:**
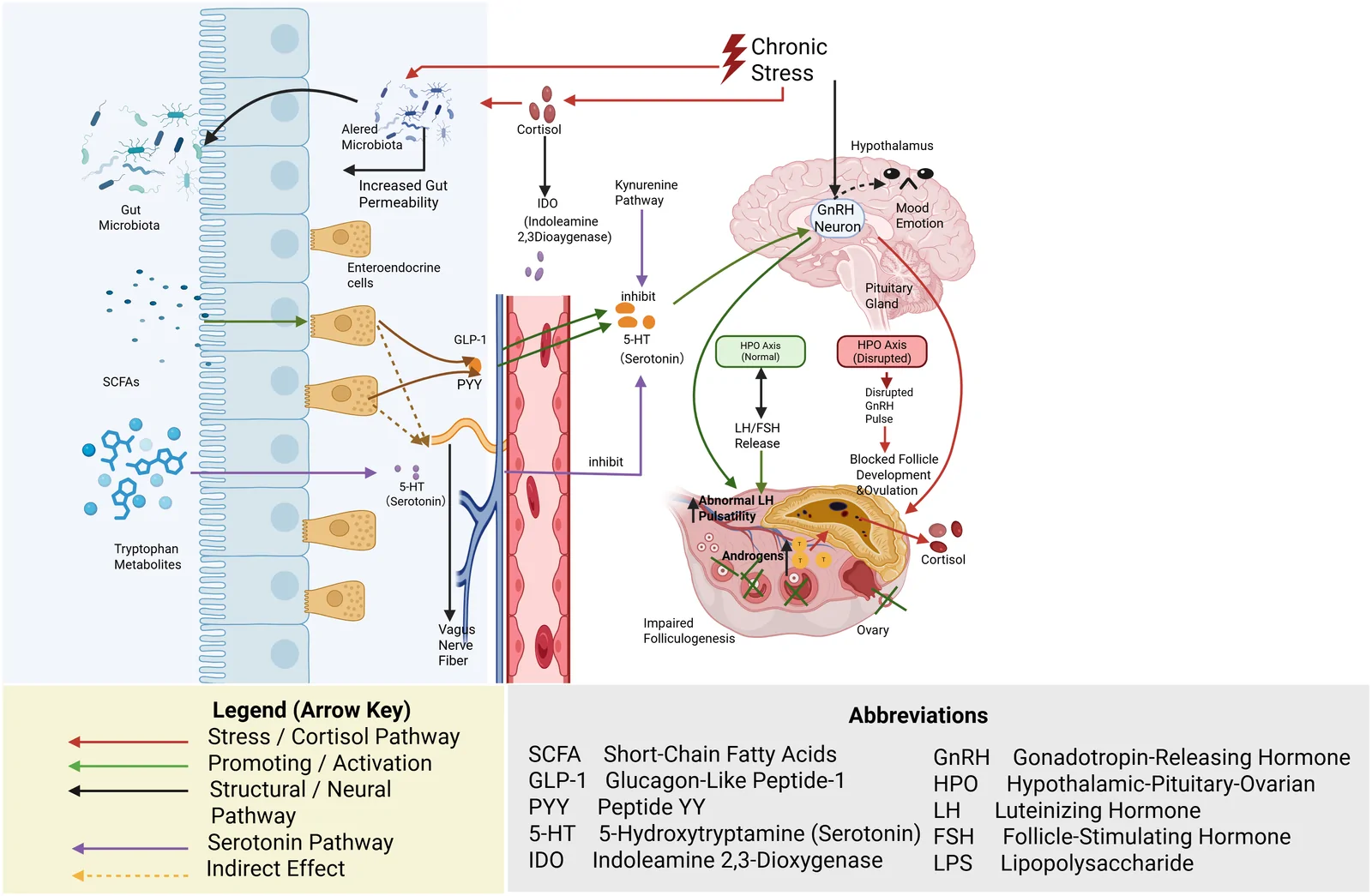
This image illustrates the gut-brain-ovary axis in the context of chronic stress. Altered gut microbiota increases gut permeability and reduces SCFA production, leading to inflammation and dysregulation of the HPO axis. Meanwhile, cortisol activates IDO, shunting tryptophan toward the kynurenine pathway and inhibiting serotonin synthesis, which further impairs GnRH pulsatility and ovarian function.

The gut is the primary peripheral site of serotonin synthesis in the human body, and its synthesis is regulated by gut microbiota. PCOS patients often exhibit alterations in tryptophan metabolism-related gut microbiota, which may affect the gut–brain circulation of 5-HT (53). Central 5-HT levels are not only associated with anxiety, depression, and other emotional comorbidities, but also act on multiple receptor subtypes in the hypothalamus to inhibit or disrupt GnRH pulsatile release, leading to abnormal LH secretion patterns that further exacerbate ovarian androgen synthesis and ovulatory dysfunction. Therefore, modulation of gut-derived 5-HT signaling may represent a potential target for ameliorating neuroendocrine imbalance in PCOS (6, 53) (**Figure 1**).

## 6 Intervention strategies

### 6.1 Dietary modification

Dietary and lifestyle interventions remain the cornerstone of PCOS management (20, 54). Dietary patterns high in dietary fiber, with low glycemic load, and containing adequate high-quality protein and unsaturated fatty acids improve insulin sensitivity, reduce chronic inflammation, and support the growth of SCFA-producing bacteria (20, 54). The Mediterranean diet, rich in dietary fiber, polyphenols, and anti-inflammatory lipids, is considered potentially beneficial for metabolic improvement in PCOS (20, 54).

Dietary approaches such as the ketogenic diet and intermittent energy restriction have shown promise for improving body weight, insulin resistance, and sex hormone profiles in small-scale studies. However, their long-term safety, reproductive benefits, and sustained effects on gut microbiota require verification in large randomized controlled trials (20, 54).

### 6.2 Probiotics, prebiotics, and synbiotics

Accumulating evidence suggests that probiotics, prebiotics, and synbiotics hold substantial therapeutic potential for ameliorating metabolic and endocrine abnormalities in polycystic ovary syndrome (PCOS) via multifaceted modulation of the gut microenvironment (55). Specifically, these interventions reshape gut microbiome structure and restore alpha diversity (microbial richness and evenness), which is consistently reduced in women with PCOS, as confirmed by a systematic review and meta-analysis of 28 clinical studies (7). Furthermore, they drive a favorable shift in the relative abundance of specific microbial taxa. For instance, supplementation with these agents generally enriches beneficial taxa, including short-chain fatty acid (SCFA)-producing bacteria (e.g., *Lactobacillus*, *Bifidobacterium*) and mucin-degrading bacteria (e.g., *Akkermansia*), while concurrently suppressing the proliferation of endotoxin-producing Gram-negative opportunistic pathogens such as *Escherichia*/*Shigella* (28). This microbial community remodeling further enhances endogenous SCFA production, preserves intestinal barrier integrity, and attenuates systemic low-grade inflammation, which collectively mediate improvements in insulin sensitivity and systemic metabolic homeostasis in PCOS patients (56).

In addition, an umbrella review of nine meta-analyses further demonstrated that probiotic supplementation significantly reduces homeostatic model assessment for insulin resistance (HOMA-IR) and fasting glucose levels, whereas synbiotic interventions exert broader beneficial effects on glycemic control, lipid profiles, and hormonal parameters (57). Nevertheless, substantial heterogeneity across existing studies—with respect to strain composition, intervention dosage, treatment duration, baseline BMI of participants, and concomitant medications—greatly limits cross-trial comparability of findings and confounds interpretation of the overall clinical efficacy (58, 59). Consequently, defining optimal strain combinations and standardized therapeutic regimens remains a major challenge. Future research should prioritize rigorous strain-level identification, precise functional validation of host–microbiome interactions, and standardized clinical outcome assessments to lay a foundation for the development of evidence-based microbiota-targeted interventions for PCOS (58).

### 6.3 Postbiotics and microbial metabolite supplementation

Postbiotics and microbial metabolite supplementation represent emerging strategies targeting the gut-ovary axis. For instance, SCFA-based interventions (e.g., butyrate) show potential for alleviating inflammation, insulin resistance, and ovarian dysfunction in animal models; specific bile acid species and AhR ligands may also exert protective effects via immune and metabolic pathways (3, 6).

However, current evidence is predominantly derived from animal models and *in vitro* experiments, with limited clinical data in humans. Therefore, postbiotics and metabolite supplementation are not yet standard of care for PCOS, and further investigation is needed to define effective doses, molecular targets, safety profiles, and long-term outcomes (6).

### 6.4 Fecal microbiota transplantation

Fecal microbiota transplantation (FMT) is a therapeutic strategy in which rigorously screened fecal microbiota from healthy donors are delivered to the recipient’s intestinal tract via oral capsules, endoscopy, nasoenteric tubes, or enema to restore intestinal microecological homeostasis. FMT has been shown to profoundly remodel recipient gut microbiota and is well established for conditions such as recurrent *Clostridioides difficile* infection; however, its application in PCOS remains largely preclinical (53, 60).

In rodent PCOS models induced by letrozole or dehydroepiandrosterone (DHEA), FMT from healthy donors reduces circulating androgens, improves estrous cycle irregularity and polycystic ovarian morphology, and partially alleviates insulin resistance and metabolic dysfunction. These phenotypic improvements are accompanied by gut microbial remodeling, including increased abundance of beneficial taxa such as *Lactobacillus*, restoration of SCFA-producing bacteria, and—in some studies—normalization of *Prevotella* (a taxon linked to PCOS dysbiosis) toward healthy control levels. Another study demonstrated that both FMT and Lactobacillus supplementation improve PCOS-like reproductive–endocrine and metabolic abnormalities via gut microbiota modulation (61). However, heterogeneity in animal models, donor sources, transplantation protocols, sampling time points, and microbiota analysis methods limits the generalizability of findings, and further validation is required (60).

### 6.5 Engineered bacteria and defined microbial consortia

Engineered bacteria and defined microbial consortia represent the future of precision microecological therapy. Theoretically, synthetic biology can generate engineered bacteria that produce specific beneficial metabolites, degrade harmful metabolites, or modulate host immune responses. However, such strategies remain preclinical for PCOS, with challenges including strain safety, controllability, colonization stability, horizontal gene transfer risk, and regulatory approval remaining to be addressed (62).

## 7 Conclusions and prospects

Accumulating evidence demonstrates that gut microbial dysbiosis and its dysregulated bioactive metabolites are critically involved in the pathogenesis of polycystic ovary syndrome (PCOS) through the gut-ovary axis. Definitive causal evidence for this axis remains largely limited to preclinical animal models and *in vitro* mechanistic studies, whereas findings from human studies are predominantly observational and correlational. This review systematically summarizes the core molecular pathways underlying the gut-ovary axis, underscoring systemic low-grade inflammation and neuroendocrine dysregulation as the two key pathological bridges linking intestinal microecological imbalance to ovarian reproductive dysfunction.

Conceptually, gut microbiota-targeted interventions represent a promising complementary strategy to conventional symptom-oriented PCOS management, yet their clinical utility requires rigorous validation in high-quality clinical studies. Currently, most mechanistic insights and emerging microecological interventions are derived primarily from preclinical *in vitro* and animal experiments, with high-level evidence from large-scale randomized controlled trials still scarce. Future research should prioritize validation and refinement of this mechanistic framework in large prospective clinical cohorts, as well as well-designed targeted interventional trials to establish safe and effective therapeutic regimens. Furthermore, dedicated investigations are warranted to delineate the precise inter-organ crosstalk between gut microbiota and the host reproductive axis, to accelerate the clinical translation of innovative microbiota-based therapies and pave the way for etiology-targeted treatment of PCOS.
